# Inclusion, characteristics and methodological limitations of systematic reviews in doctoral theses: A cross‐sectional study of all universities in Sweden

**DOI:** 10.1002/cesm.70015

**Published:** 2025-01-09

**Authors:** M. Ringsten, K. Färnqvist, M. Bruschettini, M. Johansson

**Affiliations:** ^1^ Cochrane Sweden, Department of Research, Development, Education and Innovation, Skåne University Hospital Lund University Lund Sweden; ^2^ Molecular Medicine and Surgery Karolinska Institute Stockholm Sweden; ^3^ Paediatrics, Department of Clinical Sciences Lund Skåne University Hospital Lund University Lund Sweden; ^4^ Global Center for Sustainable Healthcare, School of Public Health and Community Medicine, Sahlgrenska Academy University of Gothenburg Gothenburg Sweden

**Keywords:** doctoral thesis, quality, scoping review, systematic review, university

## Abstract

**Intro:**

A systematic review (SR) attempts to find, assess and summarize all the empirical evidence to answer a specific research question. We aim to explore to what extent reviews are included in doctoral theses from all universities with a medical faculty in Sweden, and to describe the type, topic and assess the methodological quality of the reviews.

**Methods:**

Duplicate assessors independently searched local and national repositories for doctoral theses published in 2021 within all seven medical faculties in Sweden, and categorized identified reviews based on review type, topic, and methodological quality using AMSTAR‐2.

**Results:**

5.4% (45/852) of all doctoral theses included a review, and 1.3% (45/3461) of all included studies were reviews. Of these, two thirds (31) were SRs and the rest (14) were broader ‘big picture’ reviews. The most common topics were interventions (42%) and exposure/etiology (32%), with no reviews of diagnostic tests. The majority of the SRs had very low (71%) or low (19%) quality, and few reached a high (7%) or moderate (3%) quality. The most common issues were limitations with protocols, limited search strategies, and failure to account for risk of bias in drawn conclusions.

**Conclusions:**

Few doctoral students included SRs in their theses, and the few SRs included in doctoral theses generally had a low quality. There is no consensus on the appropriate proportion of doctoral thesis including a SR. We argue that conducting a SR within a doctoral thesis can reduce redundant, harmful and unethical research, identify knowledge gaps, and help the doctoral student obtain important skills to conduct and use research.

## Plain Language summary

Systematic reviews identify, assess and synthesize research and inform decision‐making. We identified all doctoral theses published within seven Swedish universities and estimated the prevalence of any reviews, explored their characteristics and assessed their quality. Overall, only about 1 out of 100 studies were systematic reviews, and about 1 doctoral student out of 20 included any review in their thesis. The few systematic reviews included in doctoral theses generally had low quality due to issues with the application of current systematic review methods and tools. We argue that systematic reviews can help improve the relevance and quality of subsequent research and avoid research waste for doctoral students. Moreover, systematic reviews support the development of important skills for conducting and interpreting research and support reaching the educational goals of doctoral programs within medicine and health. We argue that actions are needed to support doctoral students in including and conducting high‐quality systematic reviews.

## 1 INTRODUCTION

A systematic review (SR) attempts to find, assess and summarize all the empirical evidence that fits pre‐specified eligibility criteria to answer a specific research question. SRs can be conducted within a diverse set of areas that includes, but are not limited to, interventions, diagnostic tests, prognostic models and qualitative studies. Systematic reviews are essential to ensure that decisions are informed by an up‐to‐date and complete understanding of the relevant research evidence [1]. Broader ‘big picture reviews’ are related to systematic reviews, and often have an aim to get an overarching picture of the current field of evidence. The research question at hand often determines which review should be conducted.

In 2023, approximately 6390 students were enrolled in a doctoral program in medicine and health at Swedish universities, accounting for 36% of all doctoral students across all research fields [2]. Doctoral students in Sweden are often enrolled in various specific research schools at the universities and are primarily guided by a main supervisor and one or several co‐supervisors – and committees following the progress of a doctoral student are often not the norm. The mean age to enroll as a doctoral student is 32 years, 22% of the enrolled doctoral students are not born in Sweden and 63% of the enrolled doctoral students are women as of 2023 [2]. Medical faculties in Sweden often include doctoral students from various backgrounds and fields, including medicine, biomedicine and health.

The main aim of doctoral education across all fields, which by law is needed to graduate, is to “develop the knowledge and skills required to be able to undertake autonomous research” [3]. Furthermore, specific goals must be reached within the education as determined by Swedish legislation, see Table 1. These goals overlap with the learning process, application of tools and final output of a systematic review.

**Table 1:** Learning outcomes for doctoral students, stated in The Higher Education Ordinance, Annex 2.

For the Degree of Doctor the third‐cycle student shall:
demonstrate **familiarity with research methodology** in general and the methods of the specific field of research in particular.
**demonstrate the capacity for scholarly analysis and synthesis** as well as to **review and assess new and complex phenomena**, issues and situations autonomously and **critically**
demonstrate the ability to identify and formulate issues with scholarly precision critically, autonomously and creatively, and to plan and use appropriate methods to undertake research and other qualified tasks within predetermined time frames and to **review and evaluate such work**
demonstrate through a dissertation the ability to make a **significant contribution to the formation of knowledge** through his or her own research
demonstrate the ability in both national and international contexts to present and discuss research and research findings authoritatively in speech and writing and in dialogue with the academic community and society in general
demonstrate the ability to **identify the need for further knowledge** and
demonstrate the capacity to contribute to social development and support the learning of others both through research and education and in some other qualified professional capacity.
demonstrate intellectual autonomy and disciplinary rectitude as well as the ability to make **assessments of research ethics**, and
demonstrate specialised insight into **the possibilities and limitations of research**, its role in society and the responsibility of the individual for how it is used.

## 2 OBJECTIVE

To explore the extent to which systematic reviews are included in doctoral theses from all medical faculties in Sweden, describe the type and topic and assess the methodological quality of the reviews.

## 3 MATERIALS AND METHODS

This cross‐sectional study adhered to the STROBE reporting guidance. A prespecified protocol was published before searches, data extraction, or analyses began [4], and includes additional information about the scope and methods used. Search results, all data extracted, and analyses performed are available in an open repository [4].

### 3.1 Inclusion criteria

We included all doctoral theses published in 2021 at all seven universities with a medical faculty in Sweden; Lund University; Uppsala University; Karolinska Institute; Gothenburg University; Linköping University; Umeå University; and Örebro University. Any reviews of the current literature using a systematic search and a structured research question were eligible, and the use of additional systematic methods was assessed in a separate step (see ‘Critical appraisal’‐section). This included both systematic reviews, as defined in the Cochrane Handbook [1], and reviews within the broad ‘big picture review’‐family [5].

### 3.2 Search strategy

Search strategies were developed and run by information specialists within the university library at each university from May to September, 2023. The searches and databases were tailored for each medical faculty due to differences in local repositories. Doctoral theses were available in a full‐text format for all universities. When a full‐text thesis was not available in digital format, a physical version of the thesis was identified through archives. All the identified theses with links to repositories are available in the Supplementary material.

### 3.3 Screening and data extraction

Screening and extraction forms were initially piloted for Lund University theses. Each thesis was screened for any systematic reviews independently and in duplicate in the title, abstract, the full list of conducted studies during the doctoral period, summary of the thesis content and, if needed, in full‐text. Disagreements were resolved by consensus involving a third reviewer. Any uncertainties regarding the study design in title, abstract, study list and summary‐stage of the thesis led to a full‐text assessment.

All identified reviews within the theses were screened for any separate publication in a scientific journal following the references of the systematic review within the thesis. If no reference was available within the thesis to a scientific publication, the first author's name was identified, and the publishing record of this author was further scrutinized for a scientific publication of the systematic review. More comprehensive reporting in journal articles was used, if available, in assessments of review type, topic, and methodological quality. Data extraction was done in duplicate by two independent reviewers. Any discrepancies between the two reviewers, and additional uncertainties, were resolved by discussion and consensus within the author team. Identified reviews included in doctoral theses were categorized based on “review type” (i.e., systematic reviews or broad reviews), and systematic reviews were additionally categorised based on the topic of the review. Furthermore, data were collected for how many studies were identified in the full search within the systematic reviews, number of included studies in the synthesis, software used for screening, tools used for critical appraisal and meta‐analysis, and whether the GRADE framework was used.

### 3.4 Critical appraisal

Each identified systematic review was assessed independently and in duplicate by two reviewers using the AMSTAR‐2‐tool to assess the methodological rigor for each review [6]. For non‐intervention systematic reviews where not all AMSTAR‐2 items were applicable, the tool was adapted to fit these other topics based on previous work on the adaption of AMSTAR‐2 [7], see Supplementary material for more information on how this was done. Any discrepancies between the reviewers were resolved by discussion, and consensus for judgements was reached within the author team. The methodological quality was not assessed within the broad review types due to lack of any validated instrument.

### 3.5 Outcomes

Our primary outcomes were the proportion of PhD theses, including at least one systematic review, and the proportion of systematic reviews of all articles included in the thesis. Our secondary outcomes were the type of review (systematic review and broad reviews), review category (e.g., intervention, exposure, prevalence), and the overall methodological quality of systematic review as assessed by AMSTAR‐2. A number of prespecified exploratory outcomes were also analyzed, including: the tools used to support conduction of the systematic review and time to publication from the thesis publication date. Three post‐hoc exploratory outcomes were also added during data extraction, including; the number of identified studies in the searches, the final number of included studies in the syntheses and the publication status of the reviews.

### 3.6 Statistical analysis

Descriptive statistics was used to present the primary and secondary outcomes with numbers and percentages. In addition, the exploratory outcomes also included presentation of means with standard deviation and range.

## 4 RESULTS

Our search identified 852 theses published at Medical Faculties in Sweden in 2021, including 3461 studies in total. Of these studies, 45 were reviews of any type (1.3% of all studies), and 45 doctoral theses included a review (5.3% of all theses). A flow diagram is available in the supplementary material. The proportion of reviews varied among universities, from 3.4% of all studies at Örebro University to 0% of all studies at Linköping University (Table 2).

**Table 2:** Prevalence of reviews from medical faculties at Swedish universities.

University	Proportion of studies that is a review	Proportion of theses with a review
Overall, across universities	1.3% (45 reviews of 3461 studies)	5.3%
Umeå University	2.4% (6 reviews of 246 studies)	9.8%
Karolinska Institute	1.3% (18 reviews 1347 studies)	5.3%
Lund University	0.4% (3 reviews of 696 studies)	1.8%
Göteborg University	1.8% (10 reviews of 551 studies)	7.3%
Uppsala University	1.0% (4 reviews of 385 studies)	4.3%
Örebro University	3.4% (4 reviews of 116 studies)	14.3%
Linköping University	0.0% (0 reviews of 120 studies)	0.0%

Almost all (98%; 44 out of 45) of the identified reviews were published in a scientific journal. The median number of screened records was 979 (IQR 2346) and the median number of included studies was 13 (IQR 18).

Thirty‐one (69%) of the reviews were systematic reviews according to our pre‐defined criteria, and 14 (31%) were other types of reviews (e.g., broad “big picture” reviews). Out of the 31 systematic reviews, 13 (42%) explored the effects of interventions, 10 (32%) exposure and etiology, 3 (10%) qualitative research, 2 (7%) prevalence and incidence, 2 (7%) prognostic factors and prediction models, and 1 (3%) methodology.

The broad “big picture” reviews had very broad aims and inclusion criteria, and categorizing these into distinct topic areas was not possible (all these are listed in the Supplementary material).

### 4.1 Methodological quality of the systematic reviews

The methodological quality of reviews was generally poor. Across all systematic reviews, 2 (7%) had high methodological quality and 1 (3%) had moderate. In comparison, 6 (19%) had low methodological quality and 22 (71%) had critically low with one or several critical weaknesses.

The most common methodological weaknesses contributing to lower methodological quality of the systematic reviews were issues with or lack of protocols (65%), limited search strategies (65%), and a failure to account for risk of bias in drawn conclusions (52%). All assessments and detailed justifications are reported in the Supplementary material.

### 4.2 Tools used within the systematic reviews

Most (87%; 27 of 31) systematic reviews, conducted risk of bias assessments. The most common tools were the Cochrane Risk of bias‐tools, that is, RoB1 or RoB2 (used in 39% of systematic reviews), and Newcastle‐Ottawa Quality Assessment Scales (NOQAS) (used in in 25% of systematic reviews). Five systematic reviews (15%) used the GRADE approach to assess the certainty of the evidence. The most common software used for meta‐analysis was RevMan which was used in 7 systematic reviews (35%), while 5 (25%) used Stata, and 3 (15%) used R. Five (16%) of the systematic reviews reported the use of any specific software in the screening process (e.g., such as Covidence).

Additional results for the proportion of published reviews, time to publication, use of software and specific tools in the review process are available in the Supplementary material.

## 5 DISCUSSION

We found that 1.3% of all studies included in doctoral theses from medical faculties in Sweden were reviews of any type, and 5.4% of all doctoral theses included a review. Most of the systematic reviews were of low methodological quality: only 10% had a high or moderate methodological quality.

There is no consensus on the appropriate proportion doctoral thesis including a SR. However, we argue that there are several potential benefits from increasing the proportion of high‐quality SRs in theses (Table 3). A systematic review matching the research aims of a doctoral student will identify all relevant research to inform future projects, and transparently, comprehensively and unbiased summarize the current state of knowledge. The importance of conducting a systematic review before starting new research has previously been discussed extensively [8]. For example, including a systematic review in a doctoral thesis can inform and improve the quality and relevance of subsequent studies [9]. A systematic review can also identify knowledge gaps and any ongoing studies on the same topic. This can help reduce duplication and limit redundant, harmful, and unethical research [9, 10, 11, 12, 13]. This, in turn, can limit research waste. Indeed, as much as 85% of biomedical research has been estimated to be research waste, where failure to build on current knowledge is a major contributor [14].

**Table 3:** Potential benefits of conducting a systematic review.

Identify all relevant research and thereby mitigating bias from selective outcome reporting and publication bias
Systematic mapping and creating a summary of all previous knowledge within a specific topic
Learn the strength and limitations of all previous studies within a specific topic
Learn how to critically appraise and assess uncertainty in previous studies within a specific topic
Find knowledge gaps where new research is needed – and thereby mitigating research waste and unnecessary duplication
Generate additional research ideas based on previous studies and current evidence
Identify ongoing studies, mitigating unnecessary duplication and inviting collaboration
Avoid the use of previous research without rigorous critical appraisal and assessment of risk of bias

Conducting a systematic review can also help doctoral students acquire important skills to critically consume and use research [15], identify and learn from the challenges and limitations of previous studies [16], and learn to assess publication or reporting biases [17]. At the end of the doctoral period, a systematic review can put the generated knowledge into context by relating it to all other knowledge of the same topic. A systematic review can also facilitate the translation of the findings and be used as a basis for evidence‐based decision making in healthcare, aiding in implementation or de‐implementation, and facilitating the use of the best available evidence in clinical decision making.

Not all doctoral theses benefit from including a systematic review. If there is already an available, up‐to‐date, relevant, comprehensive, high quality‐systematic review within the specific topic of the thesis, it would be a waste of resources to duplicate this effort. Indeed, with an increase in the production of systematic reviews over the last few decades, overlapping reviews have been identified as research waste [18, 19, 20]. At the same time, systematic reviews of high quality are still not the norm [19, 20] and the development of new methods for systematic reviews could lead to more accurate and nuanced results. Furthermore, as new evidence emerges, new or updated systematic reviews are needed to provide up‐to‐date evidence, and as the total number of available systematic reviews has increased, so have the available topics and types of reviews [20]. Furthermore, a systematic review should not be initiated if a broader review type fit the research questions better.

Previous methodological research has shown that different methodological choices within reviews can have a large impact on results and conclusions [21]. Replication of impactful research, such as systematic reviews, would improve, or refute the credibility of any conclusions drawn. Purposeful replication of systematic reviews is argued to play an important role in evaluating the credibility of previously generated knowledge [22] and guidance on when a systematic review should be replicated [23], updated or adapted [9] is available.

There are many potential barriers and facilitators to conducting systematic reviews within a doctoral thesis [24]. Most notably are the policy, rules and culture for including reviews within a thesis. A summary of the barriers and facilitators that we have encountered in the process of performing this study is presented in Table 4.

**Table 4:** Potential barriers and facilitators for conducting systematic reviews within doctoral theses.

**Available resources such as infrastructure, support and training. Potential activities to promote, improve or implement resources includes:**
*Courses, workshops, seminars and training in conduction of high‐quality systematic reviews and the methods that needs to be used*
*Search strategy support by trained information specialists*
*Methods support*
*Summaries and guidance to resources for systematic reviews*
*Infrastructure to support review production, e.g. Covidence for screening, RevMan Web for reporting and conducting meta‐analyses, and Cochrane Interactive Learning for training*
**The policies, cultures and mindsets in medical faculties. Potential activities to promote, improve or implement ways to change system‐related factors includes:**
*A clear policy regarding the inclusion of systematic reviews in PhD theses*
*Dissemination of policies about the inclusion of systematic reviews in PhD theses*
*Encouragement to conduct systematic reviews from supervisors, leaders of research groups and the medical faculty*
*Faculty‐wide knowledge of what a systematic review is and what it can be used for*

### 5.1 The methodological limitations in systematic review

Clear reasons for the overall subpar methodological rigor in the systematic reviews can't be determined within this study but could be due to limitations in methodological understanding within the author teams, a lack of support from methodological expertise, or simply the lack of resources needed to conduct high quality reviews within Swedish universities. Solutions on how to mitigate these issues have been previously described [25] and could be a guide for review authors.

At the same time, the presence of a small number of systematic reviews rated as having high or moderate methodological quality, without any critical flaws, shows that it is possible to create high quality systematic reviews for doctoral students and include them in a thesis. The credibility of the findings of systematic reviews has been explored in several previous studies within other samples of systematic reviews, and they generally mirror the results of this study. The rating of very low methodological quality for systematic reviews has been estimated to be 74% in reviews of low‐back pain [26], 88% within reviews of interventions after cancer [27], 88% for reviews of treatment after major depression [28], and 95% for reviews indexed in PsychINFO [29].

### 5.2 Identification of broad big picture reviews

A total of 31% of all reviews identified were broad big‐picture reviews, which was a surprisingly high proportion in light of the vague policies for what constitutes a systematic‐ and big picture review that can be included in doctoral theses across universities. This finding highlights that broader reviews fill a purpose within a doctoral thesis, are driven by the research question at hand, and that they are published in peer‐reviewed scientific journals. The policies for inclusion of different types of reviews in theses in Sweden will be explored in an upcoming project, and discussions within universities are needed to explore the current policies of broad big‐picture reviews and their inclusion in doctoral theses across universities.

### 5.3 Strengths and limitations of this study

We are not aware of any other studies exploring the extent of reviews included in doctoral theses. How Sweden compares to other countries needs to be explored further.

The main strengths of this study include the inclusion of all medical faculties within Sweden and involving collaborators from these faculties. Furthermore, the project was co‐designed, co‐executed, and facilitated by doctoral students, ensuring an end‐user perspective of the process and output. In addition, duplicate assessments and extractions, use of a pre‐registered protocol, sharing of data to facilitate reuse, and transparency enhance robustness and transparency.

A limitation of our study is that it was not possible to determine the need for a systematic review (i.e., if any systematic review was conducted outside of the doctoral theses, or if credible systematic reviews by other researchers were used as a basis for subsequent research). Nevertheless, we believe that the low proportion of systematic reviews and broader big picture reviews in doctoral theses identified in our study warrants efforts to increase this number even in the absence of such judgements. How our findings reflect the inclusion of systematic reviews in other countries than Sweden is uncertain, as there are widespread differences in policies for inclusion in theses and perceptions of systematic reviews [30].

The application of AMSTAR‐2 to interventions includes interpretation and judgment which is an inherent limitation of any critical appraisal. Within this study 6 out of 45 (13%) final methodological quality judgements differed between the two reviewers needing to be solved by discussion and consensus, which is considered to be in the higher spectrum of inter‐rater reliability [6]. Furthermore, the modification of AMSTAR‐2 to non‐intervention reviews, which it was not originally designed for, could have an impact on the final judgements – but would bias our results towards higher methodological quality and could therefore our results should be viewed as a conservative assessment. Extensions to AMSTAR‐2 for other types of studies are currently being planned [7], and the evaluation of critical appraisal tools for broad review types is ongoing [31] and could be used in updates or within similar projects.

## 6 CONCLUSIONS

A small proportion of doctoral students in Sweden conduct and include systematic reviews in their theses, and most SRs included in theses are of low methodological quality. We argue that systematic reviews can help improve the quality and relevance of subsequent primary research, and help students develop important skills. If conducting high‐quality SRs is considered an important goal in the training of the future generation of researchers—actions are needed to support doctoral students to conduct high‐quality SRs. The aim and methods used within this project could also be applied to other contexts and countries to develop a more overarching map of the extent and quality of systematic reviews in theses.

## AUTHOR CONTRIBUTIONS

**M Ringsten**: Conceptualization; data curation; formal analysis; Methodology; project administration; writing—original draft. **K Färnqvist**: Formal analysis; methodology; writing—review and editing. **M Bruschettini**: Conceptualization; formal analysis; methodology; writing—review and editing. **M Johansson**: Conceptualization; formal analysis; methodology; supervision; writing—original draft.

## CONFLICT OF INTEREST STATEMENT

MR is affiliated to Cochrane Sweden, and currently receive salary for creating and teaching related to systematic reviews in university settings, and declare no other conflicts of interest. KF is affiliated to Cochrane Sweden and declare no conflicts of interest. MJ is affiliated to Cochrane Sweden and declare no other conflicts of interest. MB is affiliated to Cochrane Sweden, and currently receive salary for creating and teaching related to systematic reviews in university settings, and declare no other conflicts of interest.

## PEER REVIEW

The peer review history for this article is available at https://www.webofscience.com/api/gateway/wos/peer-review/10.1002/cesm.70015.

## Supporting information

Supporting information.

Supporting information.

Supporting information.

## Data Availability

The data that support the findings of this study are openly available in Open Science Framework at https://doi.org/10.17605/OSF.IO/DRJ6Q and within the supplementary material files.
